# Brainstem auditory evoked potentials with speech stimulus in neonates^☆^

**DOI:** 10.1016/j.bjorl.2018.11.006

**Published:** 2018-12-29

**Authors:** Elaine Soares Monteiro Pinto, Maria Cecília Martinelli

**Affiliations:** a Universidade Federal de São Paulo, Pós-Doutorado em Distúrbios da Comunicação Humana, São Paulo, SP, Brazil; b Universidade Federal de São Paulo, Curso de Fonoaudiologia, São Paulo, SP, Brazil

**Keywords:** Child, Neonate, Auditory evoked potentials, Speech, Hearing tests, Criança, Neonato, Potenciais auditivos evocados, Fala, Testes auditivos

## Abstract

**Introduction:**

Brainstem auditory evoked potentials in response to complex sounds, such as speech sounds, investigate the neural representation of these sounds at subcortical levels, and faithfully reflect the stimulus characteristics. However, there are few studies that utilize this type of stimulus; for it to be used in clinical practice it is necessary to establish standards of normality through studies performed in different populations.

**Objective:**

To analyze the latencies and amplitudes of the waves obtained from the tracings of brainstem auditory evoked potentials using speech stimuli in Brazilian neonates with normal hearing and without auditory risk factors.

**Methods:**

21 neonates with a mean age of 9 days without risk of hearing loss and with normal results at the neonatal hearing screening were evaluated according to the Joint Committee on Infant Hearing protocols. Auditory evoked potentials were performed with speech stimuli (/da/ syllable) at the intensity of 80 dBNA and the latency and amplitude of the waves obtained were analyzed.

**Results:**

In the transient portion, we observed a 100% response rate for all analyzable waves (Wave I, Wave III, Wave V and Wave A), and these waves exhibited a latency <10 ms. In the sustained portion, Wave B was identified in 53.12% of subjects; Wave C in 75%; Wave D in 90.62%; Wave E in 96.87%; Wave F in 87.5% and Wave O was identified in 87.5% of subjects. The observed latency of these waves ranged from 11.51 ms to 52.16 ms. Greater similarity was observed for the response latencies, as well as greater amplitude variation in the studied group.

**Conclusions:**

Although the wave morphology obtained for brainstem evoked potentials with speech stimulation in neonates is quite similar to that of adults, a longer latency and greater variation in amplitude were observed in the waves analyzed.

## Resumo

**Introdução:**

Os potenciais evocados auditivos de tronco encefálico para sons complexos, como por exemplo, sons de fala, investigam a representação neural destes sons em níveis subcorticais, e refletem com fidelidade as características do estímulo. No entanto, existem ainda poucos estudos que utilizam este tipo de estímulo, e para que este possa ser utilizado na prática clínica é necessário estabelecer padrões de normalidade por meio de estudos em diferentes populações.

**Objetivo:**

Analisar, as latências e as amplitudes das ondas obtidas nos traçados dos potenciais evocados auditivos de tronco encefálico por estímulo de fala em neonatos brasileiros com audição normal e sem risco auditivo.

**Método:**

Foram avaliados 21 neonatos com idade média de 9 dias, sem risco auditivo segundo o *Joint Committe on Infant Hearing* e com resultado normal para triagem auditiva neonatal. Realizou-se potenciais evocados auditivos por estímulo de fala (sílaba /da/) na intensidade de 80 dBNA e analisou-se a latência e a amplitude das ondas obtidas.

**Resultados:**

Na porção transiente observou-se 100% de ocorrência de resposta para todas as ondas analisáveis (Onda I, Onda III, Onda V e Onda A), e este conjunto de ondas apresentou latência inferior a 10 ms. Na porção sustentada à frequência de ocorrência da Onda B foi de 53,12%; da Onda C 75%; da Onda D 90,62%; da Onda E 96,87%; da Onda F 87,5% e da Onda O 87,5% e a latência observada destas ondas variou de 11,51 ms a 52,16 ms. Observou-se maior similaridade nas latências das respostas e maior variação da amplitude no grupo estudado.

**Conclusões:**

Embora a morfologia das ondas obtidas para os potenciais evocados de tronco encefálico para o estimulo de fala em neonatos, seja bastante semelhante às dos adultos, observou-se maior latência e maior variação da amplitude das ondas analisáveis.

## Introduction

Brainstem auditory evoked potentials (BAEP) are an essential tool in audiological diagnosis, especially in young children. The electrophysiological response to stimuli such as clicks, or tone bursts is widely used in clinical practice to evaluate neural integrity and to predict auditory thresholds.^1^, ^2^

Brainstem auditory evoked potentials in response to complex sounds, such as speech sounds, investigate the neural representation of these sounds at subcortical levels, and faithfully reflect the stimulus characteristics. However, there are still few studies that use this type of stimulus, and the understanding of these auditory responses could help elucidate of the effects of age on the development of the auditory system, in addition to being useful in the evaluation of communication difficulties, learning deficits, peripheral auditory deficits, auditory neuropathies or the indication for cochlear implants.^3^

The characteristics and maturation of electrophysiological responses to speech sounds during the first year of life are still unclear.^4^ The organ of Corti develops before birth,^5^ but the maturation of the auditory pathway continues up to adolescence.^6^, ^7^

Some studies suggest that the perception of speech sounds is strongly influenced by innate factors; soon after birth neonates are able to detect differences between sounds, including sounds they have never heard before.^8^ It can also be observed that they have a similar pattern of sound perception, regardless of the language environment to which they have been previously exposed.^8^, ^9^, ^10^, ^11^ It is believed that the latency values and response morphology for speech stimuli reach the adult pattern at around 5 years of age.^12^

The auditory response evoked by speech stimulus can be subdivided into two parts:

Transient portion: constituted by the components of the onset response (beginning of the stimulus); this occurs in the first 10 ms and is similar to the response to a click stimulus, with millisecond precision.

Sustained portion: constituted by the Frequency Following Response (FFR), which occurs between 18 and 40 ms. It reflects the harmonic structure of the stimulus and provides information about the integrity of the response to the stimulus.^3^, ^13^, ^14^ The FFR is an electrophysiological measure that provides an insight into the mechanisms of subcortical processing of the stimulus in the auditory system. This term was first used by Worden and Marsh (1968) to describe a phase-locked response to the frequency components of the stimulus in cats.^15^

A study investigated the FFR characteristics in response to speech stimuli in American and Chinese neonates and adults.^16^ The authors studied the evoked potentials generated by speech stimuli according to the language (Chinese and English) and age (neonates and adults). The results showed maturation of vocal pitch processing in neonates one to three days after birth, and a significant effect of language experience in the neural processing of speech stimuli in the brainstem. These findings highlight the need to better understand neural responses in the brainstem.

As mentioned before, the perception of human speech is strongly influenced by innate factors. However, the specific language environment to which children are exposed is also crucial for the perception of speech sounds. Exposure to a particular language early in life results in a reduced ability to perceive differences between the speech sounds of other languages.^16^

Studies have shown that the brainstem auditory evoked potential with speech stimulus also showed variations in children with language disorders. A recent study^17^ investigated brainstem auditory evoked potentials in response to speech stimuli in children considered to be good readers and dyslexic children. The authors reported that children with dyslexia showed greater variability in responses than the group of children who were considered good readers. The results suggested that good readers have a stable neural representation of sound, while those who show impaired reading skills have inconsistent neural responses.

It was verified that the brainstem auditory evoked potential in response to speech stimuli may vary with age. In a study comparing responses obtained from young and old individuals,^18^ significantly higher latencies of brainstem auditory evoked potentials for speech stimuli were observed in the elderly, compared to those obtained in younger subjects, even after considering the differences in auditory thresholds between the groups. These results are consistent with reduced neural synchrony for transient speech components in the elderly.

However, the sustained portion of brainstem potentials in response to speech stimuli did not change significantly after adjustment for hearing loss, suggesting that maturity may affect response more than auditory sensitivity and other peripheral alterations.

For this potential to be used in clinical practice it is necessary to establish standards of normality through studies in populations of different age groups, exposed to different languages and without complaints.

Therefore, the aim of the present study was to analyze the latencies and amplitudes of the waves obtained from the tracings of brainstem auditory evoked potentials by speech stimuli in Brazilian neonates with normal hearing and without auditory risk factors.

## Methods

Twenty-one neonates were assessed, of whom 11 were females and 10 males, aged between 2 and 38 days of life. Only neonates without hearing risk were selected, according to the risk criteria of the Joint Committee on Infant Hearing (2007).^19^ All included subjects had a normal hearing screening, with normal results for transient evoked otoacoustic emissions and automated cortical evoked auditory potentials present at 35 dBHL.^20^ Additionally, only neonates who showed auditory evoked potentials for the click stimulus at the presentation level of 80 dBHL, with latencies within the expected for the age, were included.^1^ The present study was approved by the Research Ethics Committee (CEP 1243/11).

Brainstem auditory evoked potentials were assessed with speech stimulus during natural sleep, at the level of 80 dBHL. To undergo the test, the newborn was placed in the cradle or on the mother's lap. Three response samples were obtained, with the presentation of 1000 stimuli each.^21^ The syllable chosen for the stimulus was the synthesized syllable /da/ with a 40 milliseconds (ms) duration,^22^ available with the Navigator Pro-Biologic equipment, as proposed by Kraus et al.^23^ The sampling rate was 10.9 stimuli per second and the analysis window was 74.67 ms. The stimuli were presented through an Etymotic ER-3 Insert Earphones. After cleansing the skin with Nuprep abrasive paste, the surface electrodes were fixed with Ten20 Conductive Paste for the recording of responses, with the reference electrode being positioned on the ipsilateral earlobe, the active electrode placed on the high forehead, and the ground electrode on the low forehead. The setting included one channel. The stimuli were presented in both ears, and the starting ear (right or left) was chosen randomly.

The three tracings obtained were compared to each other and were only included if a reproducibility >60% was observed, according to the equipment software. For that purpose, we used the software tool that allows overlapping waves, performing a mathematical comparison and statistically demonstrating the correlation between them. Based on this analysis, the responses of three neonates were disregarded, as they did not show reproducibility of responses in both ears, and three ears of three other neonates were also disregarded, comprising a total of 33 valid ears. The waves were analyzed as follows:

Transient portion of the response – Peaks I, III, V and A were evaluated, determining the frequency of occurrence, latency and amplitude of each peak;

Sustained portion of the response (FFR) – Valleys B, C, D, E, F and O were evaluated by determining the frequency of occurrence, latency and amplitude of each valley.

**Figure 1:**
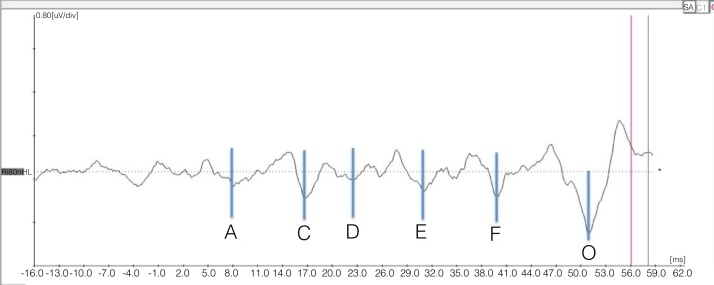
Example of the representation of valleys corresponding to the sustained portion (frequency-following response [FFR]) of the response to BAEP-speech.

In the transient portion, the wave marking occurred as it is usually done with the click stimulus, and in the sustained portion, the marking of the valleys was performed according to the baseline, beginning with valley A that follows Wave V, as shown in Fig. 1.

### Statistical method

The aim of this study was to compare quantitative variables (such as latency) using the Kolmogorov–Smirnov normality test. Student's *t* test was used for the variables with normal distribution; otherwise, the Mann–Whitney test was used to evaluate variables with non-normal distribution. Levene test was used to evaluate the significance of variance differences between groups with normal distribution. The non-parametric Kruskal–Wallis test (Kruskal–Wallis ANOVA) was also used to evaluate equality of time with the Mann–Whitney post-hoc analysis, using *α* = 0.0083 as the value for the verification of two-by-two differences.

## Results

Initially, we analyzed the occurrence of observable waves for the auditory evoked potentials for the syllable /da/ in the transient and sustained portions. In the transient portion, we observed responses in 100% of all analyzable waves (Wave I, Wave III, Wave V and Wave A). In the sustained portion, the frequency of identification of Wave B was 53.12%; of Wave C, 75%; of Wave D, 90.62%; of Wave E, 96.87%; of Wave F, 87.5%; and of Wave O, 87.5%. It was observed that all waves, except Wave B, were identifiable in >70% of subjects. However, none of the analyzed waves was present in 100% of the subjects. Table 1 shows the appearance of each of the analyzable waves for the brainstem auditory evoked potentials by speech stimulus.

**Table 1:** Occurrence of analyzable waves at the brainstem auditory evoked potentials with speech stimulus.

Wave	Occurrence
Wave I	100%
Wave III	100%
Wave V	100%
Wave A	100%
Wave B	53.12%
Wave C	75%
Wave D	90.62%
Wave E	96.87%
Wave F	87.50%
Wave O	87.50%

The latency and amplitude analyses were initially performed by separating the values found for the right and left ears. Student's *t* test was applied to evaluate whether there were statistically significant differences, and no differences were found between the ears (Table 2); therefore, the analysis was performed with the pooled values. When analyzing the response latency for the transient portion waves, it was observed that, as for the click stimulus, the group of Waves I, III and V appear in the first 10 ms after the stimulus. The latencies for the speech stimulus were higher than those expected for the click stimulus; however, the neonates’ responses were very similar.

**Table 2:** Statistical parameters – absolute latencies and amplitudes found in BAEP-speech waves in neonates, according to the ear side.

Variable	Ear	*N*	Mean	SD	LL (95% CI)	UL (95% CI)	Minimum	25th percentile	Median	75th percentile	Maximum	Kolmogorov analysis of normality	Levene's analysis of homoscedasticity	Mean difference
Correlation	**R**	17	59.59	17.56	51.07	68.11	27.00	50.50	61.00	72.00	96.00	Yes (*p* = 0.200)	Yes (*p* = 0.376)	No (*p* = 0.092)^a^
	**L**	17	69.00	13.78	62.32	75.68	36.00	63.00	68.00	83.00	90.00	Yes (*p* = 0.200)		
Click Lat. I	**R**	16	1.71	0.09	1.67	1.75	1.64	1.64	1.70	1.78	1.89	No (*p* = 0.012)	NA	No (*p* = 0.423)^b^
	**L**	16	1.68	0.05	1.66	1.70	1.64	1.64	1.67	1.70	1.78	No (*p* = 0.000)		
Click Amp. I	**R**	16	0.14	0.15	0.06	0.21	−0.14	0.04	0.11	0.25	0.47	Yes (*p* = 0.200)	Yes (*p* = 0.350)	No (*p* = 0.705)^a^
	**L**	16	0.15	0.12	0.09	0.22	−0.12	0.07	0.17	0.23	0.41	Yes (*p* = 0.092)		
Click Lat. III	**R**	16	4.45	0.16	4.37	4.53	4.26	4.32	4.42	4.54	4.76	No (*p* = 0.011)	NA	No (*p* = 0.956)^b^
	**L**	16	4.42	0.22	4.31	4.53	3.95	4.23	4.45	4.57	4.78	Yes (*p* = 0.200)		
Click Amp. III	**R**	16	0.23	0.07	0.19	0.27	0.11	0.17	0.24	0.29	0.36	Yes (*p* = 0.200)	Yes (*p* = 0.179)	No (*p* = 0.695)^a^
	**L**	16	0.24	0.09	0.20	0.28	0.10	0.20	0.22	0.29	0.46	Yes (*p* = 0.200)		
Click Lat. V	**R**	16	6.76	0.18	6.67	6.85	6.39	6.72	6.76	6.82	7.14	No (*p* = 0.013)	NA	No (*p* = 0.809)^b^
	**L**	16	6.74	0.28	6.60	6.88	6.20	6.48	6.76	6.98	7.14	Yes (*p* = 0.200)		
Click Amp. V	**R**	16	0.07	0.09	0.03	0.11	−0.09	0.01	0.07	0.13	0.22	Yes (*p* = 0.200)	Yes (*p* = 0.375)	No (*p* = 0.972)^a^
	**L**	16	0.07	0.11	0.02	0.13	−0.10	0.00	0.05	0.16	0.32	Yes (*p* = 0.200)		
Speech Lat. I	**R**	15	2.06	0.31	1.90	2.22	1.70	1.85	1.99	2.01	2.83	No (*p* = 0.000)	NA	No (*p* = 0.800)^b^
	**L**	16	1.96	0.10	1.91	2.00	1.76	1.86	1.99	1.99	2.14	No (*p* = 0.005)		
Speech Amp. I	**R**	15	0.05	0.06	0.02	0.08	−0.10	0.03	0.05	0.08	0.19	Yes (*p* = 0.200)	Yes (*p* = 0.188)	No (*p* = 0.879)^a^
	**L**	16	0.05	0.07	0.02	0.09	−0.06	0.00	0.04	0.13	0.17	Yes (*p* = 0.200)		
Speech Lat. III	**R**	15	5.16	0.52	4.89	5.43	4.62	4.89	5.07	5.32	6.82	No (*p* = 0.001)	NA	No (*p* = 0.599)^b^
	**L**	16	5.14	0.15	5.06	5.21	4.76	5.07	5.14	5.26	5.35	Yes (*p* = 0.141)		
Speech Amp III	**R**	15	0.11	0.06	0.08	0.14	−0.02	0.08	0.11	0.16	0.19	Yes (*p* = 0.200)	Yes (*p* = 0.058)	No (*p* = 0.863)^a^
	**L**	16	0.09	0.05	0.07	0.12	−0.02	0.08	0.10	0.13	0.14	Yes (*p* = 0.058)		
Speech Lat. V	**R**	15	7.68	0.42	7.46	7.89	7.24	7.39	7.64	7.83	8.95	Yes (*p* = 0.066)	Yes (*p* = 0.716)	No (*p* = 0.842)^a^
	**L**	16	7.65	0.28	7.51	7.79	7.24	7.39	7.66	7.83	8.20	Yes (*p* = 0.086)		
Speech Amp. V	**R**	15	0.04	0.05	0.01	0.07	−0.08	−0.01	0.05	0.08	0.13	Yes (*p* = 0.193)	Yes (*p* = 0.233)	No (*p* = 0.084)^a^
	**L**	16	0.01	0.04	−0.01	0.03	−0.06	−0.03	0.02	0.05	0.05	Yes (*p* = 0.172)		
Wave A Lat.	**R**	15	8.76	0.46	8.53	9.00	8.14	8.26	8.85	8.89	9.95	Yes (*p* = 0.144)	Yes (*p* = 0.424)	No (*p* = 0.660)^a^
	**L**	16	8.70	0.32	8.54	8.86	8.20	8.42	8.70	8.95	9.28	Yes (*p* = 0.200)		
Wave A Amp.	**R**	15	−0.22	0.08	−0.26	−0.18	−0.33	−0.30	−0.22	−0.17	−0.07	Yes (*p* = 0.200)	Yes (*p* = 0.101)	No (*p* = 0.720)^a^
	**L**	16	−0.23	0.05	−0.25	−0.21	−0.31	−0.26	−0.23	−0.19	−0.15	Yes (*p* = 0.200)		
Wave B Lat.	**R**	8	14.07	1.14	13.26	14.87	11.91	13.26	14.32	14.85	15.41	Yes (*p* = 0.200)	Yes (*p* = 0.231)	No (*p* = 0.450)^a^
	**L**	11	14.36	0.99	13.76	14.96	12.07	13.82	14.45	15.26	15.41	Yes (*p* = 0.200)		
Wave B Amp.	**R**	8	−0.04	0.07	−0.09	0.01	−0.16	−0.10	−0.01	0.01	0.02	No (*p* = 0.004)	NA	No (*p* = 0.206)^b^
	**L**	11	0.01	0.04	−0.01	0.04	−0.05	−0.02	0.01	0.06	0.08	Yes (*p* = 0.200)		
Wave C Lat.	**R**	12	17.69	0.87	17.18	18.19	16.72	16.91	17.67	18.16	19.64	Yes (*p* = 0.200)	NA	No (*p* = 0.887)^b^
	**L**	12	17.69	0.84	17.21	18.17	15.26	17.63	17.89	17.89	18.91	No (*p* = 0.002)		
Wave C Amp.	**R**	12	−0.12	0.15	−0.21	−0.03	−0.55	−0.13	−0.08	−0.02	−0.01	No (*p* = 0.000)	NA	No (*p* = 0.755)^b^
	**L**	12	−0.09	0.07	−0.13	−0.04	−0.25	−0.11	−0.06	−0.04	−0.01	Yes (*p* = 0.200)		
Wave D Lat.	**R**	15	22.81	1.43	22.07	23.54	20.81	22.26	22.70	22.99	26.06	No (*p* = 0.000)	NA	No (*p* = 0.051)^b^
	**L**	14	23.04	1.20	22.40	23.67	20.22	22.70	23.14	23.18	26.20	No (*p* = 0.000)		
Wave D Amp.	**R**	15	−0.31	0.18	−0.40	−0.22	−0.78	−0.40	−0.27	−0.19	−0.12	Yes (*p* = 0.190)	Yes (*p* = 0.158)	No (*p* = 0.099)^a^
	**L**	14	−0.22	0.11	−0.28	−0.16	−0.40	−0.33	−0.20	−0.15	−0.02	Yes (*p* = 0.200)		
Wave E Lat.	**R**	15	32.17	1.50	31.40	32.95	30.87	31.45	31.74	31.89	36.70	No (*p* = 0.000)	NA	No (*p* = 0.520)^b^
	**L**	16	32.27	1.39	31.57	32.96	30.72	31.49	31.77	32.15	35.54	No (*p* = 0.000)		
Wave E Amp.	**R**	15	−0.18	0.10	−0.24	−0.13	−0.35	−0.27	−0.19	−0.13	−0.01	Yes (*p* = 0.200)	No (*p* = 0.043)	No (*p* = 0.301)^b^
	**L**	16	−0.15	0.06	−0.18	−0.12	−0.27	−0.18	−0.16	−0.10	−0.05	Yes (*p* = 0.136)		
Wave F Lat.	**R**	14	40.60	1.18	39.97	41.23	38.60	40.02	40.20	41.51	42.97	No (*p* = 0.016)	NA	No (*p* = 0.839)^b^
	**L**	14	40.63	1.16	40.01	41.25	39.04	39.91	40.35	40.94	43.41	No (*p* = 0.010)		
Wave F Amp.	**R**	14	−0.31	0.30	−0.48	−0.15	−1.15	−0.30	−0.22	−0.19	0.01	No (*p* = 0.000)	NA	No (*p* = 0.150)^b^
	**L**	14	−0.20	0.14	−0.28	−0.12	−0.47	−0.26	−0.19	−0.16	0.13	Yes (*p* = 0.094)		
Wave O Lat.	**R**	15	48.49	2.17	47.37	49.61	45.16	46.77	48.22	50.12	52.16	Yes (*p* = 0.200)	NA	No (*p* = 0.650)^b^
	**L**	13	47.99	1.50	47.16	48.82	45.60	46.55	48.52	49.17	49.97	No (*p* = 0.000)		
Wave O Amp.	**R**	15	−0.38	0.77	−0.78	0.01	−3.05	−0.23	−0.16	−0.07	0.03	Yes (*p* = 0.156)	NA	No (*p* = 0.130)^b^
	**L**	13	−0.15	0.22	−0.27	−0.03	−0.83	−0.18	−0.07	−0.03	−0.02	No (*p* = 0.003)		

LL, lower limit; UL, upper limit.

a Student's *t*-test for difference of means.

b Mann–Whitney test for difference of distributions applicable to variables that did not have a normal distribution. NA, not applicable.

**Figure 2:**
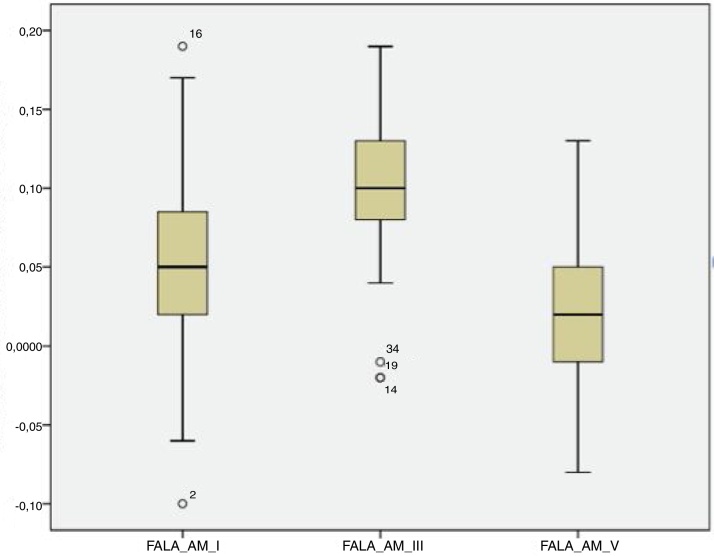
Amplitude of the waves from the transient portion to the BAEP-speech in neonates.

**Figure 3:**
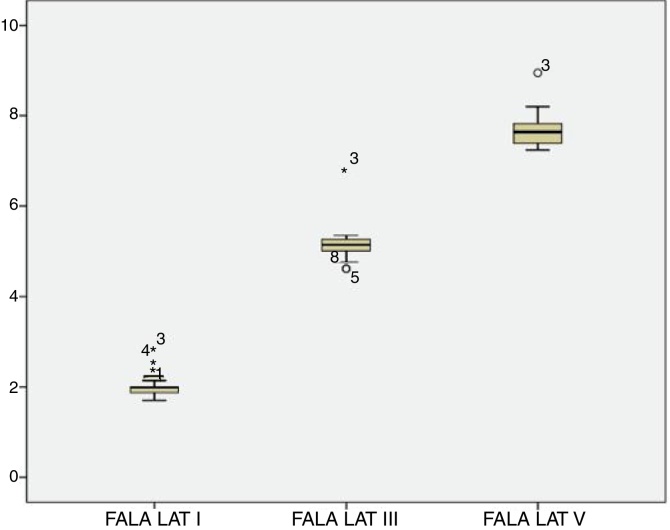
Latency of the waves from the transient portion to the BAEP-speech in neonates.

**Figure 4:**
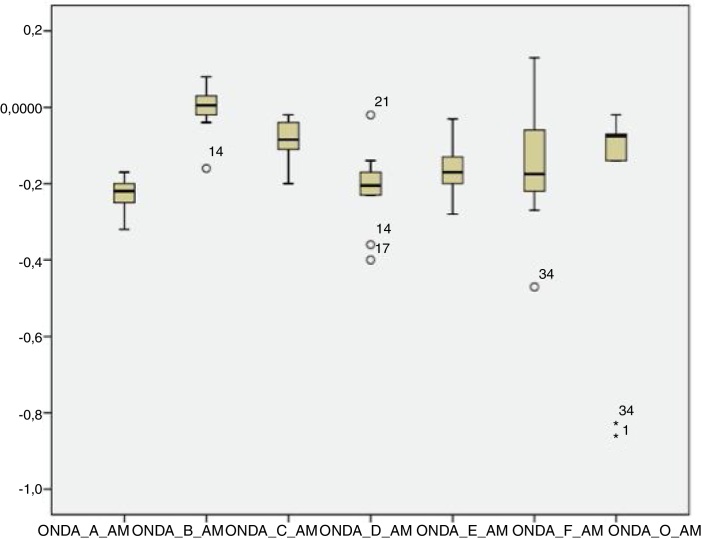
Amplitude of the waves from the sustained portion to the BAEP-speech in neonates.

**Figure 5:**
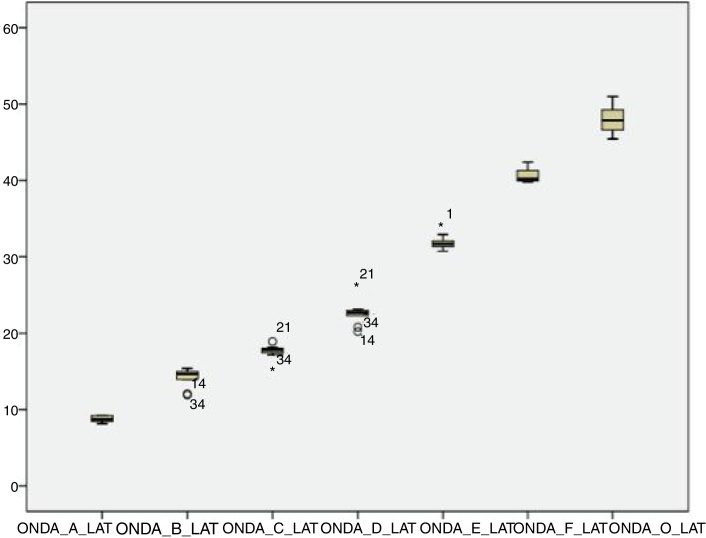
Latency of the waves from the sustained portion to the BAEP-speech in neonates.

For the click stimulus, we observed a mean latency of wave V appearance at 6.75 ms with a standard deviation of 0.23 ms, while for speech stimulus we obtained a mean latency of wave V appearance at 7.67 ms with a standard deviation of 0.35 ms. The statistical parameters of latencies and amplitudes for each analyzed wave with the grouped ears are shown in Table 3 and in Figure 2, Figure 3, Figure 4, Figure 5.

**Table 3:** Statistical parameters - Absolute latencies and amplitudes found in BAEP-speech waves in neonates.

Variable	*N*	Mean	SD	LL (95% CI)	UL (95% CI)	Minimum	25th percentile	Median	75th percentile	Maximum
Correlation	34	64.29	16.26	58.72	69.87	27	54.5	66	75.5	96
Lat. I	31	2.01	0.23	1.92	2.09	1.7	1.85	1.99	2.01	2.83
Amp. I	31	0.05	0.07	0.03	0.08	−0.1	0.02	0.05	0.09	0.19
Lat. III	31	5.15	0.37	5.01	5.28	4.62	4.95	5.14	5.26	6.82
Amp. III	31	0.1	0.05	0.08	0.12	−0.02	0.08	0.1	0.13	0.19
Lat V	31	7.67	0.35	7.54	7.79	7.24	7.39	7.64	7.83	8.95
Amp. V	31	0.02	0.05	0.01	0.04	−0.08	−0.01	0.02	0.05	0.13
Lat. A	31	8.73	0.39	8.59	8.87	8.14	8.41	8.76	8.95	9.95
Amp. A	31	−0.22	0.06	−0.25	−0.2	−0.33	−0.27	−0.22	−0.18	−0.07
Lat. B	19	14.24	1.04	13.76	14.71	11.91	13.82	14.45	14.97	15.41
Amp. B	19	−0.01	0.06	−0.04	0.02	−0.16	−0.02	0	0.02	0.08
Lat. C	24	17.69	0.84	17.35	18.03	15.26	17.23	17.89	18.03	19.64
Amp. C	24	−0.1	0.12	−0.15	−0.06	−0.55	−0.12	−0.07	−0.03	−0.01
Lat. D	29	22.92	1.3	22.43	23.4	20.22	22.63	22.7	23.14	26.2
Amp. D	29	−0.27	0.15	−0.32	−0.21	−0.78	−0.35	−0.21	−0.17	−0.02
Lat. E	31	32.22	1.42	31.71	32.73	30.72	31.45	31.74	32.04	36.7
Amp. E	31	−0.17	0.08	−0.2	−0.14	−0.35	−0.21	−0.16	−0.13	−0.01
Lat. F	28	40.62	1.15	40.18	41.05	38.6	39.95	40.2	41.18	43.41
Amp. F	28	−0.26	0.24	−0.35	−0.17	−1.15	−0.27	−0.2	−0.17	0.13
Lat. O	28	48.26	1.87	47.55	48.97	45.16	46.66	48.37	49.35	52.16
Amp. O	28	−0.27	0.58	−0.49	−0.05	−3.05	−0.22	−0.13	−0.06	0.03

LL, lower limit; UL, upper limit.

The analysis of latencies and amplitudes showed greater variation in response amplitude values than in latency. Table 2, Table 3 and Figure 2, Figure 3, Figure 4, Figure 5 show these differences. A greater similarity was observed in the latencies of responses and greater amplitude variation in the studied group.

The summary of the characteristics of the studied population and the latency results observed for each wave are shown in Table 4.

**Table 4:** Summary of the characteristics of the study population and of the latencies for each analyzed wave.

*n* = 21	Mean	SD	Interval
Gender	–	–	11 females
			10 males
Age (days)	9.11	11.55	2–38 days
Gestational age	39.26	1.17	37–41 weeks
Apgar 1st minute	8.44	1.2	5–9
Apgar 5th minute	9.67	0.49	9–10
Weight (g)	3303.06	412.06	2735.00–4265.00
Lat. Wave I (ms)	2.01	0.23	1.7–2.83
Lat. Wave III (ms)	5.25	0.37	4.62–6.82
Lat. Wave V (ms)	7.67	0.35	7.24–8.95
Lat. Wave A (ms)	8.73	0.39	8.14–9.95
Lat. Wave B (ms)	14.24	1.04	11.91–15.41
Lat. Wave C (ms)	17.69	0.84	15.26–19.64
Lat. Wave D (ms)	22.92	1.3	20.22–26.20
Lat. Wave E (ms)	32.22	1.42	30.72–36.70
Lat. Wave F (ms)	40.62	1.15	38.60–43.41
Lat. Wave O (ms)	48.26	1.87	45.16–52.16

## Discussion

### Analysis of the transient portion

The analysis of the appearance of the waves that comprise the response to the auditory evoked potentials for the syllable /da/ showed, in the transient portion (Waves I, III, V and A), 100% presence of analyzable waves. According to Table 1, it was observed that the waves appeared before 10 ms, with the average latency of Wave I = 2.01 ms, of Wave III = 5.15 ms, mean latency of Wave V = 7.67 ms and the mean latency of wave A = 8.73 ms.

Several studies^13^, ^23^, ^24^ have demonstrated that the waves of the transient portion of the response are analogous to the peaks evoked by clicks, are easily detectable with little response variability, and represents the most robust portion of the response. The onset response is a transient event that signals the beginning of the sound. In the case of consonants, the transient onset response marks the beginning of the consonant sound perception (early burst),^14^ representing the successive modulations caused by the vocal fold vibrations.^25^, ^26^

The analysis of Wave V showed a mean latency of 7.67 ms, greater than what was expected compared to that from a click stimulus. Several studies have demonstrated a higher V-wave latency for the syllable /da/ than for the click stimulus^25^, ^26^, ^27^, ^28^, ^29^ probably because the speech signal is longer and contains less high-frequency information than the click.^26^

Researchers have shown a particular interest in the wave latency differences that occur in the first 10 ms of the response (obtained by click stimulus and speech stimulus), since the portion of the neural response believed to be more congruent between the stimuli is generated in the inferior colliculus. Although there are no conclusive studies on the accuracy of neural generators for the speech stimuli, some studies highlight discrepancies in neural coding obtained by click and speech stimuli, in spite of the similar generation sites.^26^, ^28^

Authors^26^ have suggested that differences can be attributed initially to differences in the acoustic structures of the stimulus. The click is a relatively simple, non-periodic sound, with a short duration, but whose bandwidth contains a wide range of frequencies. On the other hand, the speech stimulus, in this study, the syllable /da/, begins with a relatively low-amplitude transient fast-start resources that may be especially vulnerable to background noise disturbances.

Another feasible explanation for the observed differences between the coding of the click and speech signals involves possible differences in the neural populations recruited during the two stimuli, and the findings suggest that the coding of speech sounds can recruit processes that are not present in the click coding.^26^

The transient response occurs within 10 ms after exposure to the stimulus.^24^, ^25^, ^26^ This observation was verified for the transient response (Waves I, III, V and A) in the newborns assessed in the present study.

Researchers recorded auditory evoked potentials for speech stimuli in 28 infants, aged 3–10 months, and in younger infants (3–5 months). The latency found for Wave V was 7.40 ms (in this study the mean latency of Wave V was 7.67 ms, in one-month-old neonates), and in older infants (6–10 months) the latency was 7.13 ms.^29^ Therefore, it can be observed that the latency decreases with age, probably due to the maturation process.

### Analysis of the sustained portion (FFR)

After the onset response, it is possible to analyze the sustained response consisting of the FFR. The analysis of the waves in the FFR showed that Wave B was detectable least commonly (53.12%). That result is in agreement with a study in which the authors reported that Wave B is the most inconsistent wave, and concluded that this wave could be discarded in the analysis of the Auditory Evoked Potential by speech stimulus.^23^

The other waves (C, D, E, F and O) were detected in >70% of subjects, similar to the values found by other researchers.^30^

Authors have reported that waves C and F are the most stable, with a latency standard deviation not higher than 0.5 ms in the normal population.^24^ However, the F waves occurred more frequently than the C waves among the neonates in the present study, and the standard deviation of both waves was >0.5 ms (Wave C = 0.84 ms; Wave F = 1.15 ms). However, these comparative studies were performed with children older than 8 years, suggesting that the higher latency in neonates can be explained by the incomplete maturation of their auditory systems. In a study of younger infants, Wave F latency was measured at 40.07 ms,^29^ less than that observed in this study with one-month-old infants.

Several studies have suggested that it is possible to measure FFR in neonates,^1^, ^4^, ^11^, ^31^ possibly because the peripheral structures mature earlier in relation to the central structures.^31^ The development of the auditory system involves an elaborate series of events that starts at the beginning of pregnancy and continues up to adolescence. It is assumed that this process proceeds from peripheral to central structures, with the brainstem maturing before the thalamic and cortical areas.^32^ The developmental trajectory of FFR responses to speech can be influenced by the maturity of the corticofugal pathway.^28^

Researchers have used a monosyllabic Mandarin stimulus that mimicked the English vowel /i/ and induced FFRs in American and Chinese neonates a few days after birth. The FFRs recorded in the two groups of neonates showed energy that accompanied the periodicity, such as pitch contours, of speech stimuli. It is important to note that the FFR obtained from American and Chinese neonates resembled each other and showed little differentiation.^16^ This finding provides evidence for the “biological capacity model”, indicating that neonates are born with similar innate abilities of pitch coding at the subcortical level.^15^

When studying the characteristics of FFR in neonates and adults of different linguistic origins, similar FFR is observed in neonates and adults with similar linguistic origin, despite differences in latency and amplitude. Therefore, the speech stimulus induces responses that characterize the early processing maturation in neonates, infants and children, demonstrating plasticity in the development of the auditory system in the time and frequency domains.^16^ The evaluation of these responses can help in the identification of neonates and children at risk of speech development delay and suggest preventive and therapeutic interventions for patients of all ages.^11^

The sustained FFR is synchronized with the periodicity (repetitive aspects) of the sound, with each cycle faithfully representing the temporal structure of the sound. The FFR reflects the harmonic structure of the vowel that remains during the reproduction of a periodic stimulus and shows the overall integrity of the response in relation to it.^25^, ^26^

The neural sources of FFR can be differentiated from cochlear and pre-neural cortical activity. Multiple lines of evidence strongly suggest an origin in the brainstem for the FFR recorded on the scalp. Although the onset response recorded on the scalp and FFR probably reflect multiple sources (lateral lemniscus, cochlear nucleus, lower colliculus), they provide a noninvasive method for examining the subcortical coding of speech characteristics, as well as the effect of experience on the speech resource representation.^14^

Two distinct pathways from the cochlear nucleus to the inferior colliculus were implicated in FFR generation; a direct pathway to the contralateral inferior colliculus through the lateral lemniscus, and an ipsilateral pathway via the olivocochlear system and the lateral lemniscus.^14^

### Analysis of amplitude

There was a higher variation in amplitude than in latency in both the transient and sustained portions of the waves in the present study. Some studies have shown that the latency measures provide information on the accuracy with which the brainstem responds synchronously to the acoustic stimulus, whereas the amplitude measurements provide information on the robustness with which the brainstem nuclei respond to acoustic stimulation.^21^, ^23^ Two factors can explain the amplitude variation observed in this study: the first concerns the incomplete neural maturation of the neonates; the second relates to variations in the waking state, since even though all newborns are sleeping after the examination, it cannot be guaranteed that they were asleep during data acquisition. Thus, these variations may have contributed to variations in the waveform morphology. The latencies become progressively shorter and the amplitudes of response become progressively more robust with age. Around 3–5 years of age, the values correspond to those of an adult; between 5 and 8 years of age, the latencies are even shorter and the amplitudes are more robust than those in the adult.^32^, ^33^ This event is followed by a gradual increase in latency and decrease in amplitude during adolescence until early adulthood, when the trajectory stabilizes. As of the sixth decade of life, the continuous changes in latency and frequency coding become evident once again.^32^, ^33^

## Conclusions

It was possible to observe waves with typical morphology for brainstem evoked potentials for speech stimulus in neonates. The latencies of waves I, III and V for the speech stimulus were longer than those generated by a click stimulus.

The four waves that describe the transient portion (up to 10 ms) were detected in all neonates.

The waves of the sustained portion showed rates of occurrence >70% for all waves, except for Wave B that was present in 53.12% of the neonates; the initial latency of the sustained response ranged from 10 to 52.16 ms.

The response to speech stimuli in neonates is evidence for the “biological capacity model,” indicating that neonates are born with similar innate abilities of pitch coding at the subcortical level.

## Conflicts of interest

The authors declare no conflicts of interest.
